# Complete Brachial Plexus Injury - An Amputation Dilemma. A Case Report

**DOI:** 10.5704/MOJ.1511.017

**Published:** 2015-11

**Authors:** CYL Choong, A Shalimar, S Jamari

**Affiliations:** Department of Orthopaedics & Traumatology, Universiti Kebangsaan Malaysia, Kuala Lumpur, Malaysia

**Keywords:** Flail Upper Limb, Brachial Plexus Injury, Transhumeral Amputation

## Abstract

Brachial plexus injuries with intact yet flail limb presents with problems of persistent neuropathic pain and recurrent shoulder dislocations, that render the flail limb a damn nuisance. As treating surgeons, we are faced with the dilemma of offering treatment options, bearing in mind the patient’s functional status and expectations. We present a case of a 55-year old housewife with complete brachial plexus injury begging for surgical amputation of her flail limb, 6 years post-injury. Here we discuss the outcome of transhumeral amputation and the possibility of offering early rather than delayed amputations in this group of patients.

## Introduction

Injuries to the brachial plexus present unique challenges in diagnosis and treatment to both the patient and the surgeon. The injury can be classified as complete or incomplete lesion, with either an intact or amputated limb. The intact yet flail limb presents with lack of sensation, problems of recurrent shoulder subluxation, severe pain, and is often subjected to unintentional burns and cuts. Surgeons treating such patients are often faced with the dilemma of indication and timing of elective amputation of the flail limb.

## Case Report

**Fig. 1:**
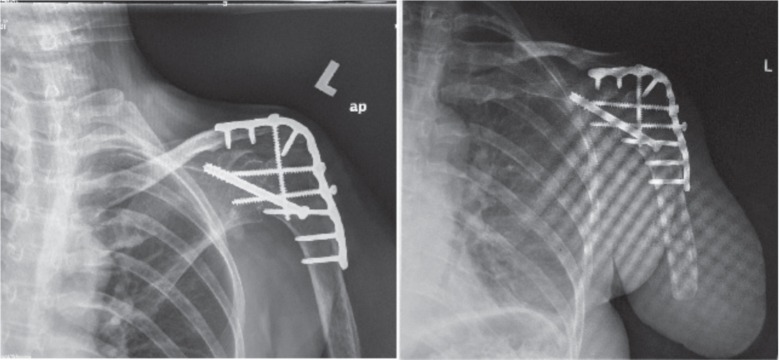
(left) – Plain AP radiographs of left shoulder pre-operatively, showing previous fusion fixation of left shoulder; (right) – Plain AP radiographs of left shoulder post trans-humeral amputation.

We present a case of a 55-year-old Chinese lady who was involved in a motor vehicle accident in 2009 and sustained a complete left brachial plexus injury. She underwent neurotization of her left upper limb 9 months post injury where her spinal accessory nerve was transferred to the suprascapular nerve, and the phrenic nerve was transferred to the musculocutaneous nerve in hope for restoration of shoulder function and elbow function respectively. Unfortunately, after a trial of neurotization, the patient clinically had shown no improvement. Over the course of 2 years, she complained of severe avulsion pain of the affected limb, developed atrophy of her deltoid, triceps, biceps and shoulder girdle musculature and experienced chronic dislocation of her left shoulder. This was followed by a left shoulder fusion surgery in 2011(Figure 1). During her clinic visits, it was noted that she continued to experience severe pain (Visual Analogue Score 8-10/10) from the preganglionic avulsion injury to her brachial plexus, despite being on several groups of analgesia. The weight of her injured limb constantly burdened her and hindered her from other daily activities. Five years after the initial injury and after 3 years of convincing the surgeon, she underwent a transhumeral amputation of the injured limb (Figure 3). Two months post-operatively, she is satisfied with the amputation because she is no longer restricted or had to carry the flail arm. She wished she had had the surgery earlier. The Disabilities of the Arm, Shoulder and Hand (DASH) questionnaire was given to her and results from the questionnaire showed a scoring of an initial 83% postoperatively and 45% after two months.

**Fig. 2:**
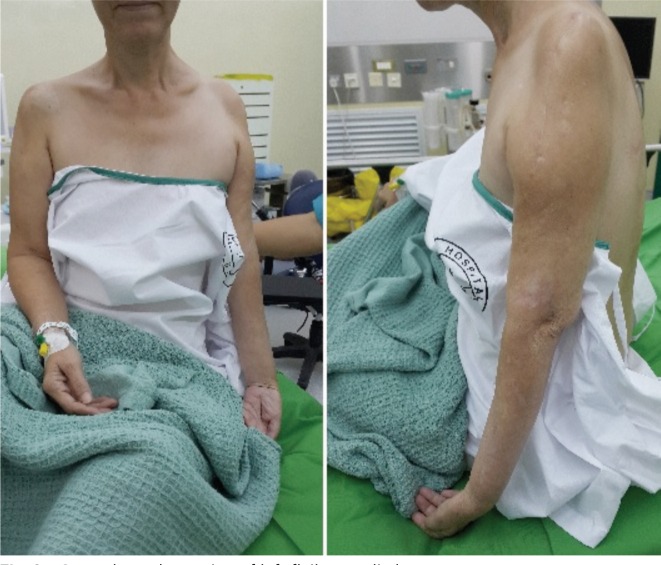
General muscle wasting of left flail upper limb.

**Fig. 3:**
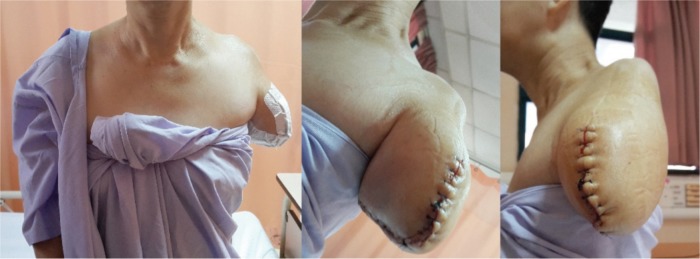
Post transhumeral amputation of the flail limb. Patient had requested the stump to be as short as possible in order for convenience of slipping on/off clothes.

## Discussion

Brachial plexus injuries invariably are traction injuries as a result of the head being forced laterally at the moment of impact or from traction across the arm^1^. When there is a complete avulsion of the brachial plexus, the choice of treatment is between three options namely, extensive surgical reconstruction of the flail limb, arthrodesis of the shoulder, or undergo amputation of the flail limb.

The decision to reconstruct or to amputate is difficult and our ability to predict when the flail limb should be electively amputated is not yet clear. Amputation is typically a choice after exhaustive salvage efforts have failed over a course of months to years. Patients with non-functional, painful, or excessively bothersome upper limbs should be offered the option of amputation.

One way to measure outcome of disability post-limb surgery is by using The Disabilities of the Arm, Shoulder and Hand (DASH) questionnaire. It is a self-report questionnaire consisting of a series of 30 questions that measure a patient’s ability to perform upper limb activities of daily living. It also allows the patient to rate symptoms, difficulty and interference with daily life according to corresponding severity levels and function levels on a 5 point Likert scale. The DASH questionnaire has good reliability with an intraclass correlation coefficient (ICC) of 0.96 and a validity of more than 0.70 on Pearson’s correlation.

In this case, the patient had the most improvement (improvement by 4 points) at two months post-operatively for recreational activities which require little effort. She had felt that there no longer was any limitation in doing regular daily activities since her amputation. However, she still had difficulties in opening a tight jar, pushing open a heavy door and placing objects above her head, changing light bulbs and carrying heavy objects weighing more than 10 lbs. The neuropathic pain of her flail limb still persisted post-amputation and this often affects her sleep.

In a study by Wilkinson *et al,* 13 out of 20 patients chose for elective amputation of their flail and useless limb. Thus, they concluded that elective amputation be performed at the patient’s request and may be considered as an element of rehabilitation. However, they noted the pain of preganglionic injury of the brachial plexus was not relieved by amputation, as was the case in our report.

Burdette *et al* suggests that a paradigm shift in limb-salvage time line should be implemented to reduce pain and suffering of patients with failed limb salvage procedures. They recommended an early delayed amputation period of 6 months post injury.

To date, there are no absolute indications that exist for amputation of upper limbs with complete brachial plexus injury.

## Conclusion

Further studies are needed to identify patients who will eventually choose early or late delayed amputation. This will enable us to provide early delayed amputation to those who desire it. By doing so, we can greatly relieve their suffering and move them more towards productive rehabilitation and independence.
